# The Prevalence and Correlation of Carotid Artery Calcifications and Dental Pulp Stones in a Saudi Arabian Population

**DOI:** 10.3390/diseases7030050

**Published:** 2019-07-15

**Authors:** Ali Alsweed, Randa Farah, Satheeshkumar PS, Rafat Farah

**Affiliations:** 1 Resident Dentist, Ministry of Health, Riyadh, Saudi Arabia; 2 Department of Internal Medicine, School of Medicine, University of Jordan, Amman 11942, Jordan; 3 Department Maxillofacial Surgery and Diagnostic Sciences, College of Dentistry, Qassim University, Al-Mulaydah, Qassim 51452, Saudi Arabia; 4 Department of Prosthodontics, College of dentistry, Qassim University, Al-Mulaydah, Qassim 51452, Saudi Arabia

**Keywords:** atherosclerosis, cardiovascular diseases, carotid arteries, dental pulp calcification, radiography

## Abstract

Background: This study sought to determine the prevalence of carotid artery calcifications (CACs) and pulp stones detected on panoramic radiographs (PRs) and ascertain their correlation. Methods: A total of 2013 digital PRs were retrospectively retrieved and thoroughly examined to determine the prevalence of CACs and pulp stones, their correlation with patient age and gender, and the relationship between the presence of pulps stones and radiographically detectable CACs. Results: The prevalence of CACs on PRs was 2.0%; the prevalence of pulp stones was 4.6%. There was no statistical relationship between pulp stones and CACs (*p* = 0.714). Older patients exhibited a significantly higher prevalence of CACs than younger patients (*p* < 0.001); pulp stones were statistically more prevalent in younger patients than older patients (*p* = 0.001). There were no significant differences between male and females in terms of the prevalence of either CACs or pulp stones (*p* = 0.087 and *p* = 0.278, respectively). Conclusions: Dentists should be trained to detect CACs on PRs belonging to patients older than 40 to exclude the presence of CACs. Moreover, pulp stones do not function as a diagnostic marker for CACs.

## 1. Introduction

Calcium mineral deposition in ectopic areas and dystrophic lesions may occur in several pathologic conditions, such as arterial atherosclerosis. This mineral deposition results in the appearance of lesions or plaque, which can be quantified radiographically. The mechanism underlying the atherosclerotic calcification represents a meeting of bone biology with chronic plaque inflammation, passive mineral deposition and/or regulated active new bone formation or remodeling. Calcification of atherosclerotic lesions occurs frequently. However, not all instances of atherosclerotic plaque calcify [1,2]. There are many reports that have correlated the presence and severity of calcifications with a patient’s total burden of atherosclerotic lesions and the increased chance of future adverse vascular events [3,4,5,6].

Panoramic radiographs (PRs) are one of the most common radiographs used in dentistry for diagnostic purposes. They contain a large amount of data captured in a single image due to their large coverage of the head and neck area. Recently, digital versions of PRs were introduced that render electronic storage and transfer of these images very easy. In addition, only a short amount of time is necessary to take these images. Additionally, their low radiation dose makes them very popular in dental clinics; almost all dental patients have a PR in their records that can be easily retrieved and reviewed on a computer screen at any time [7,8]. The carotid artery passes through an area covered by PRs; any calcifications inside the lumen of this artery can be detected as radiopacities in any high-quality PR. The ability to detect carotid artery calcifications (CACs) in PRs was first reported in the dental literature by Friedlander and Lande (1981) [9]. This subject is important owing to the high morbidity and mortality attributable to atherosclerotic lesions. Many studies have investigated the validly of radiographic quantification of CAC lesions and the prevalence of these lesions in PRs in different populations [10,11]. Researchers have also analyzed possible associations between the presence of these lesions and other cardiovascular diseases risk factors [12].

Using dental PRs to quantify CAC lesions is a cost-effective, non-invasive method that has been considered by previous studies as a valid method for this purpose [2]. Patients with radiographical signs of CACs will need a referral for more advanced diagnostic examinations such as ultrasonography, Magnetic resonance imaging (MRI), computed tomography (CT) scan, and angiography to confirm the diagnosis. In addition, suitable interventions to prevent probable future adverse vascular events will be necessary [13,14].

Among other dystrophic calcifications that occur in the head and neck region are pulp stones, which present radiographically in the coronal pulp of teeth and sometimes in the radicular pulp as a compact degenerative radiopaque mass of calcified tissues. Pulp stones are usually asymptomatic, and their pathogenies are still unknown. They are more prevalent in patients with systemic or genetic diseases such as dentine dysplasia, dentinogenesis imperfecta, and certain syndromes such as Van der Woude syndrome [15]. Idiopathic pulp stones can be detected mainly by bitewing and periapical radiographs and also by high-resolution PRs [16]. Norman and Johnstone suggested that the pathogenesis of pulp stones is the same as calcification in arteriosclerosis and that pulp stones are the local manifestation of a constitutional metabolic disturbance or dysfunction associated with the presence of a high level of blood calcium (hypercalcemia) [17,18,19]. A previous study suggested a strong correlation between the detection of pulp stones in PRs and the presence of CAC lesions; if multiple pulp stones are detected in a patient, further evaluation for CACs is required [20].

Cardiovascular diseases account for 37% of mortality due to non-communicable diseases (NCDs), which account for 73% deaths in Saudi Arabia [21]. Dentists, as medical practitioners, should contribute to reducing morbidity and mortality due to these diseases by helping with the early detection of these diseases in patients. Patients may visit a dentist before seeing their physician; increasing community awareness of these diseases and their risk factors can therefore help prevent these diseases and their complications. However, only a limited number of studies have investigated the prevalence of CACs on dental digital PRs in the adult Saudi Arabian population [22]. Therefore, this study aimed to retrospectively evaluate data from digital PRs of a Saudi Arabian population in the Qassim region to investigate the prevalence of CACs. We also investigated if there is a correlation between the presence of dental pulp stones and CACs to determine whether the presence of these stones can be used as a marker for more hazardous calcific dystrophic lesions.

## 2. Materials and Methods

### 2.1. Sample Selection

This retrospective, cross-sectional study included randomly selected digital PRs for adult patients who visited five major hospitals in the Qassim region (Qassim University Dental Hospital, King Fahad Specialist Hospital, Buraidah Central Hospital, King Saud Hospital, and Al-Rass General Hospital) for dental treatment between January 2017 and December 2017. The included digital radiographs had to be satisfactory from a diagnostic viewpoint and clearly show maxillary and mandibular teeth and the entire area posterior to the angle of the mandible at the level of the C3–C4 cervical vertebrae with optimal contrast and density and with no distortion or obscuring structure. Poor-quality radiographs with inappropriate exposure times or incorrect angulations that were non-assessable were excluded from this study. All of the digital radiographs for adult patients that satisfied the study requirements were thoroughly examined, and the patients’ demographic information—including patient gender and age—was also recorded. Patient specific data were kept anonymous. This study was conducted in full accordance with the World Medical Association Declaration of Helsinki and was approved by the Ethics Committee at the College of Dentistry, Qassim University (EA/501/2017). 

### 2.2. Radiograph Evaluation

**Figure 1:**
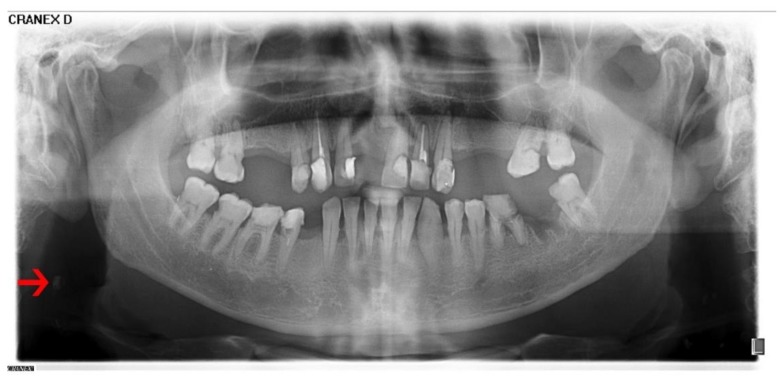
Panoramic radiograph with a detectable carotid artery calcification (red arrow).

**Figure 2:**
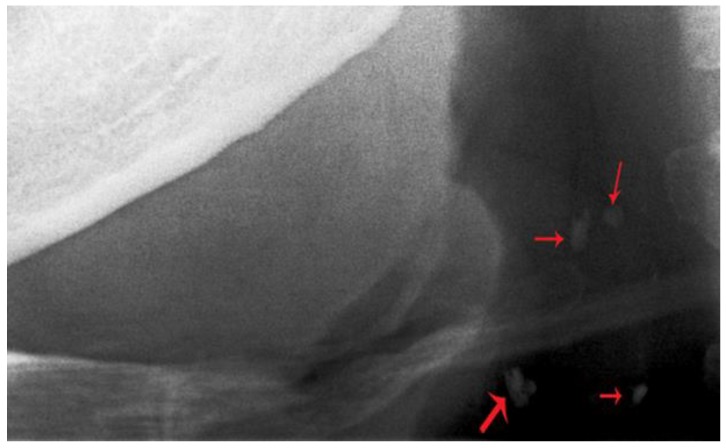
Close-up view of a panoramic radiograph showing the radio-opacities in the carotid vasculature. Red arrows indicate carotid artery calcifications.

**Figure 3:**
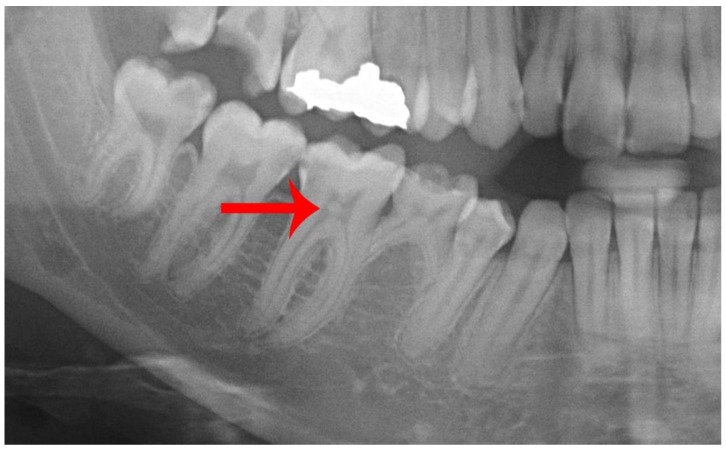
Close-up view of a panoramic radiograph showing idiopathic pulp stone within the coronal pulp chamber of the lower right first molar (Red arrow).

The included radiographs were retrieved from the hospitals’ digital archives and imported into the Qassim University College of Dentistry Radiology department’s computer system and then viewed. They were analyzed using specialized digital radiograph imaging software (DIGORA^®^ for Windows 2.7; SOREDEX, Tuusula, Finland). All of the radiographs were examined and interpreted by a single experienced oral and maxillofacial radiologist. All of the radiographs were examined on the same 21-inch liquid crystal display (LCD) monitor resolution (1920 × 1200 at 60 Hz) in a darkened room; the same ambient conditions persisted during the evaluation of all of the radiographs (Figure 1). Each original digital image was magnified using the magnification function in the software and manipulated by the investigator to enhance the contrast and brightness of the image to give the subjectively clearest image in the examined areas. The presence of CACs was diagnosed when the oral and maxillofacial radiologist observed heterogeneous unilateral or bilateral nodular, punctate, or vertico-linear radiopacities located posterio-inferior to the angle of mandible at the level of intervertebral space between the C3 and C4 vertebrae (Figure 2). The presence of idiopathic pulp stones was defined as definite radiopaque masses located within the coronal pulp chamber or in the radicular pulp canals on radiographically intact teeth (i.e., teeth without apparent fractures, caries, or fillings detected on the PR), as seen in Figure 3.

### 2.3. Statistical Analysis

All of the statistical analyses were performed using SPSS software (SPSS 20 for Windows; SPSS Inc., Chicago, IL, USA). The descriptive data are presented as frequencies and percentages. Chi-squared tests of homogeneity were used to compare differences between the male and female patients and the age groups in terms of the prevalence of both CAC and pulp stones. We also used post-hoc analysis with pairwise comparisons with the *z*-test with a Bonferroni correction (for the multinomial variable—that is, the age-sorted groups). The chi-squared test of independence and Fisher’s exact test (when the expected cell frequencies were less than 5) and the phi (φ) coefficient were used to test the association between pulp stones and CACs. We adopted a statistical significance level of *p* < 0.05.

## 3. Results 

The PRs of 2013 patients were included and analyzed in this study. The mean age of the patients was 34.3 ± 13.9 years (range: 18–77 years); 1212 (60.2%) patients in the cohort were male, and 801 (39.8%) were female. Seven hundred and twelve patients (35.4%) were younger than 25, 624 (31.0%) patients were aged 26–40, 473 (23.5%) patients were aged 41–54, and 204 (10.1%) patients were older than 55. 

We found 41 CACs in the 2013 PRs (2.0%). Of these CACs, 30 were in male patients and 11 were in female patients. There was no statistically significant difference between the male and female patients in terms of the prevalence of CACs (*p* = 0.087). There was a moderately strong statistically significant association between the patients’ age and the presence of CACs (*p* < 0.001). Post-hoc analysis involving pairwise comparisons using the *z*-test of two proportions with a Bonferroni correction revealed that the prevalence of radiographically detectable CACs in the two younger age groups (18–25 and 26–40) was as not statistically significantly different. There were statistically significantly differences between the 40–54-year age group and the greater-than-55 age group in terms of the prevalence of CACs. Additionally, there were significant differences between the older group and both younger groups (Table 1).

The prevalence of pulp stones was 4.6%, and there was no statistically significant difference in prevalence between the male and female patients (*p* = 0.278). There was a statistically significant association between the patients’ age and the presence of CACs (*p* = 0.001). The detectable pulp stones were observed more frequently in younger age groups compared with the older age groups (Table 2).

We used Fisher’s exact test to investigate the association between CACs and pulp stones because there was one expected cell frequency less than five. We failed to find a statistically significant association (*p* = 0.714). There was a very weak association between CACs and pulp stones (φ = 0.002, *p* = 0.937) (Table 3). 

## 4. Discussion

We investigated the prevalence of CACs detected on digital PRs in a Saudi Arabian population. The use of PRs to detect CACs has been reported in many studies as a valid tool. These studies reported a very high and positive association between PRs and ultrasonography and Doppler sonography in the detection of CACs [4,11,23]. The presence of CACs in PRs is also associated with carotid stenosis and the resistive index calculated from duplex sonography [10]. Furthermore, many studies have supported the notion that the presence of CACs in PRs is considered a risk indictor of significant peripheral arterial disease and cardiovascular disease, such as myocardial infarction and coronary heart disease events, stroke, transient ischemic attack, and metabolic syndrome [24]. Here, we attempted to establish a relationship between the presence of dental pulp stones and CACs. 

Differential diagnosis of CACs in PRs is not an easy task for clinicians. In addition to requiring deep knowledge of neck anatomy, a clinician needs to know and be able to differentiate CAC lesions in PRs from other similar anatomical or pathogenic radiopacities that may be present in the submandibular and cervical region (e.g., calcified triticeous and thyroid cartilages, hyoid bone, calcified lymph nodes, phleboliths, and submandibular salivary gland sialoliths) [14]. In this study, an oral and maxillofacial radiologist examined high-quality PRs using specialized software that afforded the ability to manipulate the contrast, brightness, and magnification of an image. This process enhanced the ability of the radiologist to correctly interpret the calcific lesions and therefore increased the validity of the study results.

The prevalence of CAC lesions in previous cross-sectional studies ranged from 2–6%, which differed according to the study population’s age, gender, ethnicity, and lifestyle [25,26,27,28]. The reported CAC prevalence was much higher when the study population included diabetic patients (26–36%) or patients with severe carotid stenosis (84%) [4,29]. The prevalence of CACs in randomly selected PRs of adult patients was 2.0% in this study, which is at the lowest end noted in the literature, and lower than the 5% prevalence reported in a Saudi population by Alzoman et al. [22]. This fact may be due to Alzoman et al. studying the prevalence of CACs in patients 30 years and older; in this study, the population consisted of all adult patients (18 years and older). Our study population was mostly young adults, which reflects the population distribution in Saudi Arabia, a country composed of mostly young adults. Older patients (55 and older) represent only around 9% of the population in Saudi Arabia [30].

We found a non-significant difference in the prevalence of CACs between males and females, however, the prevalence in males was higher than that in females. These findings contradict the results of Nasseh and Georges [31] and Santos et al. [27]. These authors found a higher prevalence of CACs in females. However, it is important to point out that CACs and cardiovascular disease in general are more prevalent in post-menopausal females and that the presence of CACs in these patients has been reported to be associated with severe abdominal aortic calcification and a higher risk of future vascular incidents [32]. In this study, nearly 81% of CACs detected in female patients were present in females older than 40 years; the prevalence of CACs in the female old-age group (≥55 years) was 7%. In general, CAC lesions occurred more frequently in older age groups for both male and female patients; the prevalence of CACs was as high as 12.7% in patients older than 55. This finding is consistent with previous studies and the fact that atherosclerosis is prevalent in older individuals [26,27]. 

Pulp stones were detected in 93 cases out of a total of 2013 examined radiographs (i.e., a prevalence of 4.6%). This prevalence is much lower than the prevalence previously reported in Indian and Turkish populations [33,34,35]. Furthermore, the occurrence of pulp stones in this study population was higher in younger patients, which contradicts the findings of many previous studies. This result may be justified by the fact that most of screened radiographs for older patients contained many carious, missing, and filled teeth with many root canal treatments. The prevalence of decayed, missing, and filled teeth (DMFT) among older age groups in Saudi Arabia has been reported in previous studies to be up to 24.3, which justifies the lower prevalence of idiopathic pulp stones in this age group [36,37]. 

Data pertaining to the location (maxillary and mandibular, and anterior or posterior) and the teeth involved in the pulp stone were not recorded because the aim of this study was to consider the presence of these stones as a diagnostic marker for other potential medical issues . There was no correlation between the presence of pulp stones and CACs. On the contrary, only two male patients, both older than 55, had both pulp stones and CACs detected in their panoramic radiographs. The remaining pulp stones were detected in patients without CAC lesions. This result is similar to the conclusions of previous studies; the presence of pulp calcification was not a strong predictor for the presence of CACs [38,39]. Yeluri et al. [20] found a significant correlation between dental pulp stones, carotid artery calcifications, and renal calcifications. The presence of a large number of pulp stone in young patients in this study and previous clinical reports confirming the finding of these calcifications in young permanent teeth may indicate that the formation of these stones is linked to genetic or developmental causes [15,16]. Carotid artery calcifications, on the other hand, are caused by degenerative processes [1]. This difference in the underlying mechanism of pathogenies may justify the weak correlation between pulp stones and carotid calcifications.

Panoramic radiographs can detect only calcified atherosclerotic plaques, which are considered to be more stable compared with less calcified or non-calcified plaque. These latter forms of plaque have been reported to be more inflamed and prone to detachment and thrombosis formation [1]. Plaque calcification occurs late in the course of atherosclerotic lesions, which results in late diagnoses in PRs. Not all CAC lesions detected in radiographs are positively diagnosed as true CACs due to misdiagnoses with other radiopaque structures in the region [14]. Nevertheless, detection of these calcified silent lesions by a dentist on an X-ray should be considered seriously, as it indicates a serious underlying cardiovascular disease and is considered to be a quantitative marker for cerebrovascular ischemic event risk and legal liabilities. A detection can help patients become aware of the potential risk. Additional referrals and interventions after a detection of CACs in dental radiographs may prevent future serious cerebrovascular and cardiovascular adverse events, which can be life threating and may lead to serious disabilities. 

Despite the limitations and inherent drawbacks of any observational cross-sectional study, there is always a need for follow-up with a prospective study. This study emphasizes the role of the dentist as part of the medical team, not only in the promotion of oral health but also as a first line of defense for the detection of diseases that may jeopardize a patient’s general health and lead to serious future disability or even death. Patients may visit a dentist before visiting a physician. The results of this study urge dentists to be aware of these lesions. Dentists can also enroll in training workshops to enhance their abilities to detect these lesions. Furthermore, dentists can examine patients’ radiographs—particularly those of individuals older than 40—for the presence of CACs. Until now, there has been no dental marker (e.g., dental pulp stones) that indicates the presence of artery calcification. The detection of CACs on PRs, followed by prompt referral for further care in positive cases, could potentially decrease morbidity and mortality due to cardiovascular diseases and cerebrovascular incidents.

## 5. Conclusions

The radiographic prevalence of CACs in this Saudi Arabian population was 2.0%, and CACs were significantly more frequent in patients older than 40. There was no statistically significant difference in CAC prevalence as a function of gender. The prevalence of pulp stones was 4.6%, and they were more common in younger patients. Their presence in a radiograph was not a marker for the existence of CACs in patients. Therefore, this study suggests that dentists should be trained to exam all PRs that belong to patients ≥40 years old to exclude the presence of CACs in these patients. 

## Figures and Tables

**Table 1:** Descriptive analysis of study variables and the relation to carotid calcifications (CACs) (*n* = 2013).

Variables	Category	Total Number	Percentage %	CACs			*p* Value
				Absent	Present	Percentage %	
Gender	Male	1212	60.2%	1182	30	2.5%	0.087
	Female	801	39.8%	790	11	1.4%	
Age group	18–25	712	35.4%	712	0	0%	<0.001 *
	26–40	624	31.0%	622	2	0.3%	
	41–54	473	23.5%	460	13	2.7%	
	≥55	204	10.1	178	26	12.7%	

* indicates statistically significant difference (*p* < 0.05).

**Table 2:** Descriptive analysis of study variables and the relation to pulp stones (*n* = 2013).

Variables	Category	Total Number	Percentage %	Pulp Stones			*p* Value
				Absent	Present	Percentage %	
Gender	Male	1212	60.2%	1151	61	5%	0.278
	Female	801	39.8%	769	32	4%	
Age group	18–25	712	35.4%	663	49	6.9%	0.001 *
	26–40	624	31.0%	596	28	4.5%	
	41–54	473	23.5%	462	11	2.3%	
	≥55	204	10.1%	199	5	2.5%	

* indicates statistically significant difference (*p* < 0.05).

**Table 3:** Descriptive analysis of study variables and the association between pulp stones and carotid calcifications (CACs) (*n* = 2013).

Variables	Category	Total Number	CACs/Pulp Stones			
			−*/*−	+/−	−/+	+/+
Gender	Male	1212	1123	28	59	2
	Female	801	758	11	32	0
Age group	18–25	712	663	0	49	0
	26–40	624	594	2	28	0
	41–54	473	449	13	11	0
	≥55	204	175	24	3	2
